# Symphysiotomy in Zimbabwe; Postoperative Outcome, Width of the Symphysis Joint, and Knowledge, Attitudes and Practice among Doctors and Midwives

**DOI:** 10.1371/journal.pone.0003317

**Published:** 2008-10-10

**Authors:** Hege Langli Ersdal, Douwe A. A. Verkuyl, Kenneth Björklund, Staffan Bergström

**Affiliations:** 1 Division of International Health (IHCAR), Karolinska Institutet, Stockholm, Sweden; 2 Department of Obstetrics and Gynaecology, United Bulawayo Hospital, Bulawayo, Zimbabwe; University of Cape Town, South Africa

## Abstract

**Background:**

Obstructed labour remains one of the leading causes of maternal and foetal death and morbidity in poorly resourced areas of the world, where the 24 hours availability of a caesarean section (CS) cannot be guaranteed, and the CS related mortality rate is still high. In these settings, reinstatement of symphysiotomy has been advocated. The objectives were, in1994; to study perioperative and long-term complications of symphysiotomy and compare them to those related to CS for similar indications, in 1996; to measure the symphyseal width after symphysiotomy and compare it to that after normal vaginal delivery, and, in 1998; to assess knowledge, attitudes and practice related to symphysiotomy among doctors and midwives in Zimbabwe.

**Methods and Findings:**

Thirty-four women who had undergone symphysiotomy and 29 women who had undergone a CS for obstructed labour were interviewed. The symphyseal widths of 19 women with a previous symphysiotomy were compared to that of 92 women with previous normal vaginal deliveries, using ultrasound technique.

Forty-one doctors and 39 midwives, in three central hospitals and seven district hospitals in Zimbabwe, were interviewed about symphysiotomy. None of the 34 women reported serious soft tissue injuries or infections post symphysiotomy. Long-term complications after symphysiotomy do not differ notably from those after CS for similar indications. The intra-articular width of the symphysis pubis is increased after a symphysiotomy. Seventy-nine of the 80 interviewed health care workers knew about symphysiotomy. One obstetrician had performed symphysiotomies. Two-thirds of the participants considered symphysiotomy an obsolete and second-class operation, but lifesaving and appropriate in remote areas of Zimbabwe. Ten of 13 midwives in remote areas wanted to carry out symphysiotomies themselves.

**Conclusions:**

No severe complications due to symphysiotomy were revealed in this study. The results suggest that a modest permanent enlargement of the pelvis post symphysiotomy (together with the absence of a scarred uterus) may facilitate subsequent vaginal delivery. Doctors and midwives working in district hospitals have a more positive attitude to symphysiotomies than the colleagues in central hospitals. Obstetricians (who would have to do the teaching), working in the large urban hospitals almost exclude symphysiotomy as an alternative management in Zimbabwe.

## Introduction

Symphysiotomy was developed in Europe late in the 18^th^ century as a means to save the lives of mother and child in obstructed labour. In the early days the separation of the bones was performed after exposing the symphysis. With the introduction of aseptic techniques in the mid 19^th^ century and the subcutaneous symphysiotomy by Frank [1], using local anaesthetics, the operation gained acceptance and became part of the obstetric arsenal in many parts of Europe [2]–[5]. The technique was further refined by Zárate in Argentina [6], and symphysiotomy was extensively practised in Latin America and Ireland up to the mid 20^th^ century [4], [7], [8]. Seedat and Crichton in Durban, South Africa, further developed the technique [9] and symphysiotomy came to be used in Africa for the ensuing decades [9]–[12]. Currently, outside Africa, symphysiotomies are still performed in Papua New Guinea [13].

Mechanical obstruction in the second stage complicates 1–2% of labours [14]. In developing countries this percentage might be higher because women often do not attain their (pelvic) growth potential due to stunting. The facilities and staffing level of non-private hospitals in developing countries make it often inevitable that CSs are associated with an appreciable mortality rate. In addition, there is no guarantee that a woman with a scar in the uterus can, or even would like to, reach in time a (still adequately staffed) hospital for the next delivery. The World Health Organization (WHO) estimates annual global obstructed labour related maternal mortality at 50,000 [15]. On top of that, prolonged labours frequently cause life long handicaps to mother and child. According to the United Nations Population Fund (UNFPA) there are 2 million women living with a vesico-vaginal fistula caused by obstructed labour (http://www.endfistula.com/fast_facts.htm). The woman in obstructed labour should not be abandoned in her plight.

Symphysiotomy is a potential solution in selected cases of obstructed labour with mild to moderate cephalopelvic disproportion [12], [16]–[18]. A review of 5,000 symphysiotomies performed during the twentieth century concluded that: “If valid conclusions can be drawn from one hundred years of retrospective studies, there is considerable evidence to support a reinstatement of symphysiotomy in the obstetric arsenal, for the benefit of women in obstructed labour and their offspring” [16]. A theoretical model shows that under basic circumstances only a few symphysiotomies are needed to prevent one maternal death [19]. Still, symphysiotomies are rarely performed systematically, with notable exceptions [17], [20]. In contrast, Onah demonstrated a dislike for CS among pregnant Nigerian women [21], and a preference for symphysiotomy above CS, if given the choice [22].

In view of the ongoing controversy we found reasons to contribute to the discussion with a rare long term case control follow up study. At the same time we evaluate for the first time symphyseal width after symphysiotomy and after normal vaginal delivery with ultrasound technique, and knowledge, attitudes and practice related to symphysiotomy among doctors and midwives in Zimbabwe.

## Methods

The present paper builds on two follow-up studies post symphysiotomy performed in Bulawayo, Zimbabwe in1994 and 1996 and one study in 1998 on knowledge, attitudes and practice among doctors and midwives at three levels of the health system in Zimbabwe; the academic hospital in Harare (Harare Central Hospital), two central hospitals in Bulawayo (United Bulawayo Hospital and Mpilo Central Hospital) and a convenience sample of seven district hospitals (Beitbridge, Lupane, Esigodini, Gwanda, Hwange, Victoria Falls and Kariba).

### Follow-up post symphysiotomy, studies in 1994 and 1996

Permission to conduct the interviews and ultrasound examinations, including ethical clearance, was obtained from the medical superintendents at United Bulawayo Hospitals (UBH) and Mpilo Hospital. All women were informed about the research project (in English or a local language) when seen by the first author (HLE) together with a local interpreter or an ultrasound technician. Since these follow-up studies were conducted among a partly illiterate population the women gave explicit verbal consent to involvement. Nobody refused. The interpreter or the ultrasound technician bore witness to the oral consent process.

In 1994, 18 women who had undergone a symphysiotomy previously (S group) and 29 women with previous CS (CS group) were interviewed.

The second author (DAAV) had made a serious effort to record basic data and the addresses of all the women who had a symphysiotomy in the UBH and the referring Matabeleland South Province district hospitals during 1990–1994. Also women who had a symphysiotomy performed even earlier and who were seen incidentally at the antenatal clinic or in labour were added to the list. Altogether 61 women were registered. Symphysiotomies had mostly been performed by consultants in UBH, and by experienced district doctors who considered symphysiotomy to be the best option in each of the cases.

The CS group consisted of women having undergone caesarean delivery for a probable pelvic outlet obstruction. They were recorded in the register as having had a CS after a failed vacuum extraction. It was not possible to find enough time-matched failed vacuum extractions followed by CS in the registers of the UBH, therefore we included the delivery records of the other tertiary hospital in Bulawayo, Mpilo Hospital, which caters to similar women. Seventy women were selected in this way and we tried to match the time since the index delivery as much as possible.

Serious efforts were made to visit women in both groups. Fourteen women in the S group and 24 women in the CS group were found at home. Letters written in local languages and English, promising a check up and expressing commitment to pay travelling costs and compensation (for themselves and necessary accompanying person), were sent to the remainder. After this, four and five women, respectively, appeared.

The first author (HLE) prepared the interview in cooperation with DAAV. HLE conducted all the interviews and took notes, assisted by a single interpreter speaking fluent English, and the local languages.

The interview was divided in four sections. Firstly, the baseline data (age, parity before and after index intervention, and years since index intervention) were confirmed and registered. Secondly, the woman was asked about the child's condition when born and subjectively experienced perioperative complications (pain, fistulae, laceration, hemorrhage or infection). Thirdly, she was questioned about specific current complaints (pain when walking, dancing, jumping or carrying, painful scar, dyspareunia, infertility, and incontinence), and finally, the women were asked about any subsequent pregnancies and deliveries.

In 1996, 19 women who had undergone a symphysiotomy previously (S group) and 92 women with only previous normal vaginal deliveries (NVD group) were interviewed and the symphyseal joints were examined by ultrasound at UBH. Ethical clearance for this study was also given in advance by The National Committees for Research Ethics in Norway.

The register of women who had undergone symphysiotomy in the area around Bulawayo had in the mean time expanded to 70. Letters were written, inviting them to UBH for a checkup. Nineteen women appeared representing the S group, three of whom had also been interviewed in 1994. Two of these 19 women were pregnant (both 8 months). Eleven women lived in and around Bulawayo, four lived 20–100 kilometres outside the city, and four lived over 100 kilometres away.

At the Department of Obstetrics and Gynaecology in UBH, 92 women with previous normal vaginal deliveries, referred for ultrasonography for different reasons, were during one month consecutively invited to have their symphyseal width recorded. Further inclusion criteria were parity 1–8, having delivered exclusively vaginally, age between 15 and 45 years, and no orthopaedic disorders. All the selected women were informed about the study, and all of them accepted to participate. Thirty-four of the women included were pregnant.

Baseline data for both groups were registered and women in the S group were interviewed by HLE using the same semi-structured interview as in 1994. There was always an ultrasound technician present who spoke the local languages and English. Afterwards, women in both groups were sonographically examined by HLE with a Philips Sterling 1993 model, utilizing 5 mega Hertz convex sector probe to measure the internal symphyseal gap. Björklund et al had concluded in 1996 [23] that estimating the width of the symphysis pubis by ultrasonography offers at least the same precision as radiography. The image of the symphysis is seen as two curved lines representing the anterior surfaces of the pubic bones, turning parallel into the symphyseal gap, where the width may be measured. Measuring the width may sometimes be difficult due to the shape of the gap. The opposing edges of the gap might be highly irregular and there is a widening mostly upwards, but also occasionally downwards. The measuring point was defined as the narrowest part of the symphyseal joint gap in the upper half of the symphysis. The women were in a supine position with extended hips while the measurements were taken.

For statistic calculation Student's t-test was used.

### Knowledge, attitudes and practice among doctors and midwives, study in 1998

The interviews were not announced beforehand. All relevant doctors and midwives present at the respective maternity units at the time of the visit were invited to participate, and everyone accepted. Permission to conduct the interviews, including ethical clearance, was obtained from the medical superintendents at each location.

The first and second authors (HLE and DAAV) prepared the questions in cooperation with a local general practitioner in Bulawayo. The interview consisted of two parts. Firstly, the participant was asked about her/his practice and if he/she could describe the procedure. Secondly, the interview included a series of statements on symphysiotomy. The person interviewed was asked to react affirmative or negative to closed questions. HLE conducted all the interviews.

In the data analysis the interview locations were divided into urban and rural, comparing the responses of specialists in obstetrics and gynaecology, junior doctors and district doctors and different categories of midwives.

## Results

### Follow-up post symphysiotomy, studies in 1994 and 1996

#### Baseline data

The baseline data from the three groups (S, CS, and NVD) are presented in Table 1. Twelve women in the S group had delivered before their symphysiotomy, five with a CS. Women in the NVD group had no abdominal deliveries, and their average parity when seen was 2.6.

**Table 1:** Baseline data from the three groups; CS, S, and NVD.

	CS 1994		S 1994+1996		NVD 1996	
	n = 29		n = 34		n = 92	
	Average	Range	Average	Range	Average	Range
Maternal age	26,8	15–40	26,1	18–42	31,1	19–45
Parity before index operation	1	0–5	0,8	0–6		
Interval since operation, years	2,9	0–15	4	0–15		
Total number of deliveries at follow up	2,3	1–6	2,5	1–8	2,6	1–8

#### Reported perioperative complications after symphysiotomy

There was one neonatal death during the hospital admission associated with a symphysiotomy among the 70 deliveries on record.

None of the 34 interviewed women in the S group reported serious soft tissue injuries in the birth canal, e.g. laceration, fistulae, and hemorrhage, or post-operative infection. One woman delivered a baby in a district hospital, the neonatal death, with an undiagnosed hydrocephalus and experienced a too wide separation of the symphysis resulting in damage to the tissues supporting the urethra/bladder neck. She suffered from stress incontinence as soon as the urinary catheter had been removed. Thirteen (38%) of the women did not remember their symphysiotomy delivery as being particularly painful.

#### Current complaints

Table 2 shows a comparison of current complaints in the S and CS groups.

**Table 2:** Comparison of current complaints after CS and after S.

	CS	S	P
	n = 29	n = 34	
	%	%	
Pain when walking*	13,8	23,5	0,33
Pain when dancing	6,9	2,9	0,52
Pain when jumping	6,9	8,8	0,78
Pain when carrying	6,9	8,8	0,84
Painful scar	51,7	2,9	<0,01
Dyspareunia	17,2	29,4**	0,41
Infertility	6,9	0	0,12
Incontinence	6,9	2,9	0,47

* CS 4/4 pain when walking any distance.

S 1/8 pain when walking any distance, 7/8 pain when walking long distance.

** 10/10 some pain over symphysis pubis when abduction of the legs.

The outcomes are very similar for the two interventions, except for scar pain (p<0.01).

Eight women (23.5%) in the S group reported that they felt pain when walking; seven of them had tenderness or pain over the symphysis pubis only after walking 10–20 km. The woman who delivered a baby with hydrocephalus reported pain in the symphysis pubis and a feeling of instability in the pelvis when walking any distance. She was the only woman in the S group with urinary stress incontinence at follow up. She could presently cope with her incontinence if she remembered to void frequently. She managed her daily duties, but was not able to walk far from the village. None of the other women in the S group experienced instability in the symphysis pubis or the pelvic region.

Half of the women in the CS group experienced pain in the scar. They also mentioned itching, pins and needles. Five women in the CS group reported pain deep in vagina during coitus, none of them having had this problem before the CS. Ten women in the S group reported some pain over the symphysis pubis with abduction of the legs during coitus, but none experienced pain in vagina. The women were not asked about possible psychosocial complications arising from this.

#### Width of symphysis pubis and subsequent deliveries

Table 3 shows the width of the symphysis pubis in women post symphysiotomy and in women with only previous normal vaginal deliveries. The average symphyseal width after symphysiotomy was 9.2 mm. Among women with previous normal vaginal deliveries the average distance was 4.7 mm (p<0.01). The outcome of subsequent deliveries after CS and after symphysiotomy is presented in table 4.

**Table 3:** The width of symphysis pubis after S and after NVD.

	S			NVD			P
	n	Average	Range	n	Average	Range	
		mm	mm		mm	mm	
Not pregnant	17	9,2	2,0–20,4	58	4,7	1,9–7,8	<0,01
Pregnant	2	13,9	12,8–15,0	34	5,8	2,9–9,7	<0,01

**Table 4:** Outcome of subsequent deliveries after CS and S.

	CS		S	
	n	%	n	%
Total number of women	29		34	
No of women with subsequent delivery	8	27,6	16	47,1
Total number of deliveries	10		22	
Normal vaginal deliveries	6	60	19	86,4
Caesarean section	4	40	3	13,6

### Knowledge, attitudes and practice among doctors and midwives, study in 1998

Eighty doctors and midwives were invited to participate in this study, and all accepted. The interviews in the urban settings comprised 34 doctors and 26 midwives, and in the rural settings 7 doctors and 13 midwives. Thus a total of 41 doctors and 39 midwives were interviewed. Of the doctors in the urban settings, 10 were obstetricians and 24 junior doctors. In the district hospitals, all the 7 doctors interviewed were non-specialist. Of the midwives in the urban settings, 13 were clinical instructors, 10 qualified midwives and 3 student midwives. In the rural settings, 4 were clinical instructors, 7 were qualified midwives and 2 were student midwives.

Seventy-nine of the 80 participants knew about symphysiotomy, and 76 could describe the technique, including 16 of the 17 midwife instructors. One junior doctor was not aware of the intervention. One of the ten obstetricians had occasionally performed a symphysiotomy the other nine did not practice the intervention, but indicated that they would be able to carry it out. Among the 24 junior doctors in the urban settings, six wanted to learn the skill. One of the seven district doctors thought he could do a symphysiotomy if need be, another two wanted to learn the procedure.

Table 5 shows the responses of the doctors to a series of statements on symphysiotomy, comparing the opinions of obstetricians and junior doctors working in the large cities with those of district doctors. The latter have more positive attitudes to symphysiotomy. Table 6 compares the responses of doctors and midwives in the urban and rural settings, respectively, to the same series of statements as those of Table 5. All the rural midwives (n = 13) regarded symphysiotomy as a lifesaving operation appropriate for remote areas, and 23 of the 39 midwives (59%) thought that the procedure should be taught to midwives. One third of the doctors and two thirds of the midwives thought that symphysiotomy should be performed, if only for teaching purposes, in central hospitals.

**Table 5:** Affirmative answers on statements about symphysiotomy among obstetricians and junior doctors in three hospitals in the two largest cities of Zimbabwe; Harare and Bulawayo, and among district doctors at seven district hospitals.

	Urban		Rural
	Obstetricians	Junior doctors	District doctors
	n = 10 (%)	n = 24 (%)	n = 7 (%)
Symphysiotomy			
is a harmful operation	6 (60)	16 (67)	2 (28)
is obsolete and second-class	8 (80)	16 (67)	4 (57)
can be lifesaving	3 (30)	12 (50)	5 (71)
is appropriate in very* remote areas	5 (50)	9 (37)	5 (71)
Symphysiotomy has			
negligible maternal mortality	8 (80)	18 (75)	6 (86)
high maternal morbidity	8 (80)	15 (63)	2 (28)
uneventful subsequent deliveries	7 (70)	5 (21)	2 (28)
Symphysiotomy should be			
performed in central hospitals	2 (20)	9 (37)	3 (43)
taught to district doctors	2 (20)	9 (37)	4 (57)
taught to midwives	2 (20)	5 (21)	2 (28)

* More remote areas than existing in Zimbabwe1998.

**Table 6:** Affirmative answers on statements about symphysiotomy among doctors and midwives in three hospitals in the two largest cities of Zimbabwe (Harare and Bulawayo), and among doctors and midwives at seven district hospitals.

	Urban		Rural	
	Doctors	Midwives	Doctors	Midwives
	n = 34 (%)	n = 26 (%)	n = 7 (%)	n = 13 (%)
Symphysiotomy				
is a harmful operation	22 (65)	11 (42)	2 (29)	4 (31)
is obsolete and second-class	24 (71)	17 (65)	4 (57)	9 (69)
can be lifesaving	14 (41)	20 (77)	5 (71)	13 (100)
is appropriate in remote areas	14 (41)	20 (77)	5 (71)	13 (100)
Symphysiotomy is associated with				
negligible maternal mortality	26 (76)	10 (38)	6 (86)	4 (31)
high maternal morbidity	23 (68)	11 (42)	2 (29)	4 (31)
uneventful subsequent deliveries	12 (35)	12 (46)	2 (29)	10 (77)
Symphysiotomy should be				
performed in central hospitals	11 (32)	15 (58)	3 (43)	9 (69)
taught to district doctors	11 (32)	20 (77)	4 (57)	11 (85)
taught to midwives	7 (21)	13 (50)	2 (29)	10 (77)

Eight of the ten obstetricians stated that their opinions on symphysiotomy were based on what they were taught to believe in medical school or during specialist training, and that they had not been influenced by papers on symphysiotomy they had read later in their careers. Three of the ten obstetricians thought that randomised controlled trials of symphysiotomies were needed, whereas the other seven saw no need for further research into a procedure they regarded as outdated. Ninety percent of the junior doctors indicated that their opinion on symphysiotomy was based on what they were taught in medical school. One of the 39 midwives was aware of scientific studies on symphysiotomy, the rest had never heard of any such studies. Fifty-one percent of the doctors spontaneously commented that symphysiotomy had been abandoned in Western countries, and considered this fact an argument against the use of the intervention in Zimbabwe.

## Discussion

There are no reports of maternal mortality directly related to symphysiotomy from the antibiotic era [16]. There are still large regional differences in CS-related mortality. A study from the adjacent Matabeleland-South province of 1,128 CSs performed in 7 district hospitals 1998–2000 (covering a period when the health/transport infrastructure was still good) revealed a CS-related mortality of 1.6% [24]. In circumstances where CSs are not very dangerous and where women have few children (hence few deliveries after a previous CS) the advantages of symphysiotomies are small and furthermore doctors cannot obtain or maintain the expertise needed [19], but symphysiotomies are still sometimes the best option even in this situation [25]–[29]. Verkuyl calculated that in breech presentations in district hospitals in sub-Saharan Africa a policy of replacing elective CS with trial of labour combined with a symphysiotomy if the foetal head became stuck, would, in nullipara, prevent one maternal death for two symphysiotomies performed [19]. Replacing emergency CS for failed vacuum extraction with a symphysiotomy was claimed in this paper to have a similar beneficial effect on maternal mortality [19].

Hill et al estimated 535,900 maternal deaths worldwide in 2005 [30], of which 50% were concentrated in sub-Saharan Africa and there was no significant reduction in maternal mortality ratios between 1990 and 2005 in this area. For all other countries with data there was a decrease in maternal mortality ratios of 2.5% per year in the same time period [30]. Symphysiotomy is an alternative management in cases of mild to moderate cephalopelvic disproportion, live foetus and longitudinal lie. The British Journal of Obstetrics and Gynaecology proclaimed, in an editorial (2002), the rebirth of symphysiotomy to reduce maternal and foetal mortality and morbidity [31].

According to the literature symphysiotomy is associated with immediate post-operative pain and discomfort [4], [10], [12], [13], [16]–[18], [32]–[38], and it is a painful procedure when insufficient local anaesthesia is given. All women will experience pain at the moment of delivery, and separating the different origins of pain in labour might be difficult, also because an episiotomy and mostly a vacuum extraction is part of the symphysiotomy procedure.

The results from the present study and previous follow-up studies indicate that symphysiotomy confers an acceptable level of complaints in the long run [10], [16], [32], [36], [38]. Lasbrey assessed pain in the symphysis pubis, groin, hip, thigh, sacro-iliac joint and stress incontinence, mostly associated with physical activity, and found that these problems were small and did not interfere with day-to-day activities [36]. The prevalence of these symptoms at some time during the follow-up period or in a subsequent pregnancy was 58% in the symphysiotomy group and 60% in parous women with unassisted vaginal deliveries. Hartfield compared the outcomes of symphysiotomy and CS (for cephalopelvic disproportion) including a long-term follow-up study [10]. Prevalence figures of reported sub fertility (7%), stress incontinence (3%), and backache (25%) in the symphysiotomy group were similar to corresponding figures in the CS group. Infertility after symphysiotomy was not addressed in the present study, and the woman who reported incontinence was apparently subject to an incorrect judgement of the degree of cephalopelvic disproportion. Pain or tenderness over the symphysis pubis when walking long distances was observed in this study, but not reported nor mentioned as a parameter in previous follow-up studies [4], [10], [13], [32]–[38]. Dyspareunia was registered in 17,2% (CS group) and 29,4% (S group) of the women in this study, unfortunately women with normal vaginal deliveries were not asked about this. Overall, prevalence of maternal complaints after symphysiotomy and CS do not differ much, though they are somewhat different in nature.

The study performed in 1996 indicates that the width of symphysis pubis is permanently increased after a symphysiotomy, potentially facilitating future vaginal deliveries. Over three-quarters of the women with previous symphysiotomy that had a subsequent delivery had given birth vaginally without complications, in agreement with previous observations [3]–[5], [20], [25], [27]. This is an important advantage for a young woman with a borderline pelvis and several pregnancies ahead, living in a rural area with a tradition to deliver at home far from a well equipped hospital.

The studied S group, including almost 50% of the women recorded after a symphysiotomy, might be a biased sample. Maybe women with severe complications were kept away, or the other way around, women without complaints had little to gain from leaving home for 1–2 days. Health services were free and well organized at that time, so it is likely to believe that the network of clinics would have picked up a woman with severe handicap and referred her. All the women in the record were operated in Bulawayo or an adjacent mission hospital, and the registered addresses were in the same region. Both postal service and bus transport system were reliable in 1994 and 1996.

Doctors and midwives, working in Zimbabwe in 1998, knew in theory what a symphysiotomy is, and the majority of the participants in this study considered the operation lifesaving and appropriate in remote areas. Knowledge, attitudes and practice may have changed with time, but the results from 1998 indicate that district doctors and midwives have a more positive attitude to symphysiotomy than colleagues working in the cities. Most positive are the midwives working in rural areas. They have to manage every day with poor medical facilities and limited resources, including transport. These midwives often see mothers and their babies in bad condition due to prolonged obstructed labour, and frequently have to handle the situation alone. Ten of 13 district midwives wanted to learn how to perform a symphysiotomy themselves, in agreement with an editorial in the British Journal of Obstetrics and Gynaecology of 2002: “Symphysiotomy is simple. ….It requires only local anaesthesia, a catheter, a scalpel and a pair of gloves. It can be performed safely and successfully by individuals who are not obstetricians” [31]. The technique utilized does not require special surgical skills, and experience indicates that symphysiotomy may be carried out by a well-trained midwife if training is provided by an experienced obstetrician [24], [32].

On the other hand, the obstetricians are the most reluctant subgroup, 90% had never done a symphysiotomy, not even on a model like their colleagues in the United Kingdom have to do as part of their specialist training, to be able to handle emergency situations. The obstetricians are responsible for the education of new doctors and they will also influence the teaching of student midwives. Their lectures are of great importance because only a few of the participants in this study had access or paid attention to the scientific literature on symphysiotomy.

The present situation in Zimbabwe (like in several other countries) demonstrates another important factor related to symphysiotomy. In the wake of the economical collapse, a previously well organised health service struggles to maintain standards. Midwives and doctors emigrate. Transferring patients is often not possible because fuel is unobtainable. Women with a scar of previous CS(s) are in mortal danger when pregnant. Some of them could have been delivered in the past by a symphysiotomy, and that would have prevented the scar and made her pelvis somewhat larger.

### Conclusion

The frequency and severity of long term complications post symphysiotomy do not differ from those after CS. The permanent increase of the width of the symphysis pubis, measured in this study, seems to facilitate subsequent vaginal deliveries. Therefore, the benefit of symphysiotomy in cases of mild to moderate cephalopelvic disproportion is positively correlated to the local risks of a CS (nearly always the alternative course of action), the number of deliveries expected in the future, and the chance that subsequent deliveries would not happen in a well equipped and staffed hospital. This study indicates that almost 50% of doctors and 85% of midwives working in a developing country agree with this statement.
